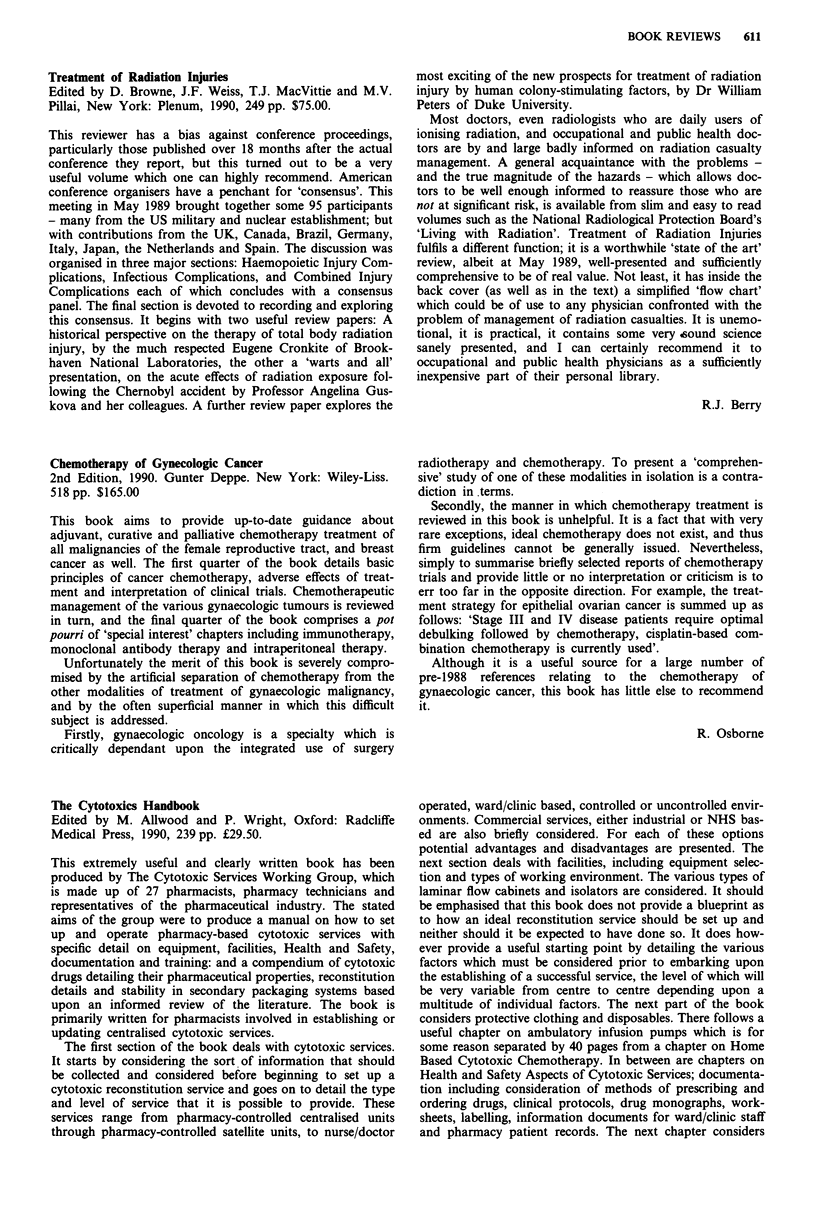# Treatment of Radiation Injuries

**Published:** 1991-09

**Authors:** R.J. Berry


**Treatment of Radiation Injuries**
Edited by D. Browne, J.F. Weiss, T.J. MacVittie and M.V. Pillai, New York: Plenum, 1990, 249 pp. $75.00.

This reviewer has a bias against conference proceedings, particularly those published over 18 months after the actual conference they report, but this turned out to be a very useful volume which one can highly recommend. American conference organisers have a penchant for 'consensus'. This meeting in May 1989 brought together some 95 participants – many from the US military and nuclear establishment; but with contributions from the UK, Canada, Brazil, Germany, Italy, Japan, the Netherlands and Spain. The discussion was organised in three major sections: Haemopoietic Injury Complications, Infectious Complications, and Combined Injury Complications each of which concludes with a consensus panel. The final section is devoted to recording and exploring this consensus. It begins with two useful review papers: A historical perspective on the therapy of total body radiation injury, by the much respected Eugene Cronkite of Brookhaven National Laboratories, the other a 'warts and all' presentation, on the acute effects of radiation exposure following the Chernobyl accident by Professor Angelina Guskova and her colleagues. A further review paper explores the most exciting of the new prospects for treatment of radiation injury by human colony-stimulating factors, by Dr William Peters of Duke University.

Most doctors, even radiologists who are daily users of ionising radiation, and occupational and public health doctors are by and large badly informed on radiation casualty management. A general acquaintance with the problems – and the true magnitude of the hazards – which allows doctors to be well enough informed to reassure those who are *not* at significant risk, is available from slim and easy to read volumes such as the National Radiological Protection Board's 'Living with Radiation'. Treatment of Radiation Injuries fulfils a different function; it is a worthwhile 'state of the art' review, albeit at May 1989, well-presented and sufficiently comprehensive to be of real value. Not least, it has inside the back cover (as well as in the text) a simplified 'flow chart' which could be of use to any physician confronted with the problem of management of radiation casualties. It is unemotional, it is practical, it contains some very sound science sanely presented, and I can certainly recommend it to occupational and public health physicians as a sufficiently inexpensive part of their personal library.

R.J. Berry